# Serum reference value of two potential doping candidates—myostatin and insulin-like growth factor-I in the healthy young male

**DOI:** 10.1186/s12970-016-0160-9

**Published:** 2017-01-05

**Authors:** Der-Sheng Han, Chi-Huang Huang, Ssu-Yuan Chen, Wei-Shiung Yang

**Affiliations:** 1 Department of Physical Medicine and Rehabilitation, National Taiwan University Hospital BeiHu Branch, Taipei, Taiwan; 2 Community and Geriatric Medicine Research Center, National Taiwan University Hospital BeiHu Branch, Taipei, Taiwan; 3 Department of Athletic Training and Health, National Taiwan Sport University, TaoYuan, Taiwan; 4 Department of Physical Medicine and Rehabilitation, National Taiwan University Hospital and National Taiwan University College of Medicine, No. 7, Chung-Shan South Rd., Taipei, Taiwan; 5 Department of Internal Medicine, National Taiwan University Hospital, No. 7, Chung-Shan South Rd., Taipei, 100 Taiwan; 6 Graduate Institute of Clinical Medicine, College of Medicine, National Taiwan University, Taipei, Taiwan; 7 Center for Developmental Biology & Regenerative Medicine, National Taiwan University, Taipei, Taiwan; 8 Department of Physical Medicine & Rehabilitation, National Taiwan University Hospital Yunlin Branch, Yunlin, Taiwan

**Keywords:** Biomarker, Doping, Exercise, Reference value, Fat-free mass

## Abstract

**Background:**

Myostatin negatively regulates muscle growth, and its inhibition by suitable proteins can increase muscle bulk and exercise performance. However, the reference values of serum myostatin in athletes performing strength training are still lacking.

**Methods:**

A cross-sectional study recruiting28 male collegiate athletes performing strength training and 29 age-matched normal controls was conducted.

The serum concentration of myostatin and insulin-like growth factor 1 (IGF-1), grip strength, and body composition were the main outcome measures. We used regression models to analyze the correlation between serum markers and the physiological parameters. The athlete group had greater height, weight, body mass index (BMI), fat mass percentage, fat-free mass, muscle mass, waist girth, grip strength, and estimated daily energy expenditure.

**Results:**

The IGF-1 concentration was higher in the athlete group (324 ± 80 vs. 263 ± 134 ng/ml), but the myostatin levels did not differ (12.1 ± 3.7 vs. 12.4 ± 3.5 ng/ml). The reference value for IGF-1 among the healthy young males was 293 ± 114 ng/ml, correlated with age and height; the value for myostatin was 12.3 ± 3.6 ng/ml, correlated negatively with BMI, fat mass percentage, and waist girth after adjustment for age.

**Conclusion:**

Myostatin level is negatively related to fat percentage, and serum IGF-1 is positively related to height. The reference values could provide a basis for future doping-related study.

## Background

As a member of the transforming growth factor β superfamily, myostatin is a negative regulator of muscular growth and differentiation. Myostatin knock-out mice have three times more muscle mass than their wild-type counterparts [1]. Better functional performance is also observed without noticeable physiological compromise in these animals. On the other hand, nude mice over-expressing myostatin showed a cachectic phenotype with both skeletal muscle and adipose tissue atrophy [2]. Myostatin is translated into a 310-amino acid peptide, and cleaved as N terminal propeptide and C terminal active peptide. It is then circulated in the blood as an endocrine hormone [2]. The N-terminal propeptide is a potent inhibitor for myostatin. In a human study, a male baby born to a professional athlete mother in Germany was found to have a bulky muscle mass and loss-of-function myostatin mutation [3]. The serum myostatin peptide level was as low as undetectable in this male baby. It is reasonable to hypothesize that inhibiting myostatin can increase muscle mass and strength, and the sports performance of athletes. Another study revealing that the serum myostatin propeptide concentration in a bodybuilder was significantly higher than that in an untrained volunteer supports this hypothesis [4]. Strength training, a mode of exercise that can increase muscle mass, can modulate myostatin. Strength training inhibits intramuscular myostatin mRNA and protein expression in rats [5]. In a human study, similar findings were reported by Roth et al. that muscular myostatin mRNA level decreased 37% in healthy volunteers after a 9-week course of heavy unilateral knee extension strength training [6]. In addition, Walker et al. claimed that the serum myostatin peptide concentration, analyzed with Western blot, decreased 20% in a healthy male volunteer after a 10-week whole body strength training course [7].

Insulin-like growth factor-1 (IGF-1), a downstream molecule of the growth hormone (GH), increases protein synthesis in skeletal muscle. IGF-1 transgenic mice have accelerated skeletal muscle regeneration. The hypertrophic effects of IGF-I infusion on muscle are well documented in animal models and muscle cell culture systems. [8, 9] IGF-1 expression increased in a rat model of cardiomyocyte hypertrophy and in healthy volunteers doing strengthening exercises [10]. Using the C2C12 myotube model, a dose-dependent reduction in myostatin expression was observed on exposure to GH; this action was reversed by the GH antagonist pegvisomant [11]. We previously proposed that myostatin and IGF-1 could serve as a brake and accelerator mechanism for regulating muscle mass or function [12]. Based on this accelerator-brake model, we hypothesized that both IGF-1 and myostatin may correlate with lean or fat-free mass.

Due to their ability to modulate muscle mass and function, myostatin and IGF-1 genes and their protein products might be a target for doping [13]. Myostatin-inhibiting antibody and muscle-specific expression of IGF-I were shown to increase the muscle mass and function of *mdx* mice, an animal model of Duchenne muscular dystrophy [14, 15]. It is reasonable to expect improved muscle function after injection of exogenous IGF-1, myostatin propeptide, or myostatin neutralizing antibody, which will change their serum concentrations. However, we still do not have their reference value in the general population or in the athlete. Therefore, we conducted a cross-sectional study to investigate the reference values of serum myostatin and IGF-1 concentrations in male student athletes and untrained counterparts, and secondarily to determine whether relationships with body composition exist for each serum marker in the grouped cohort.

## Methods

### Participants

**Fig. 1:**
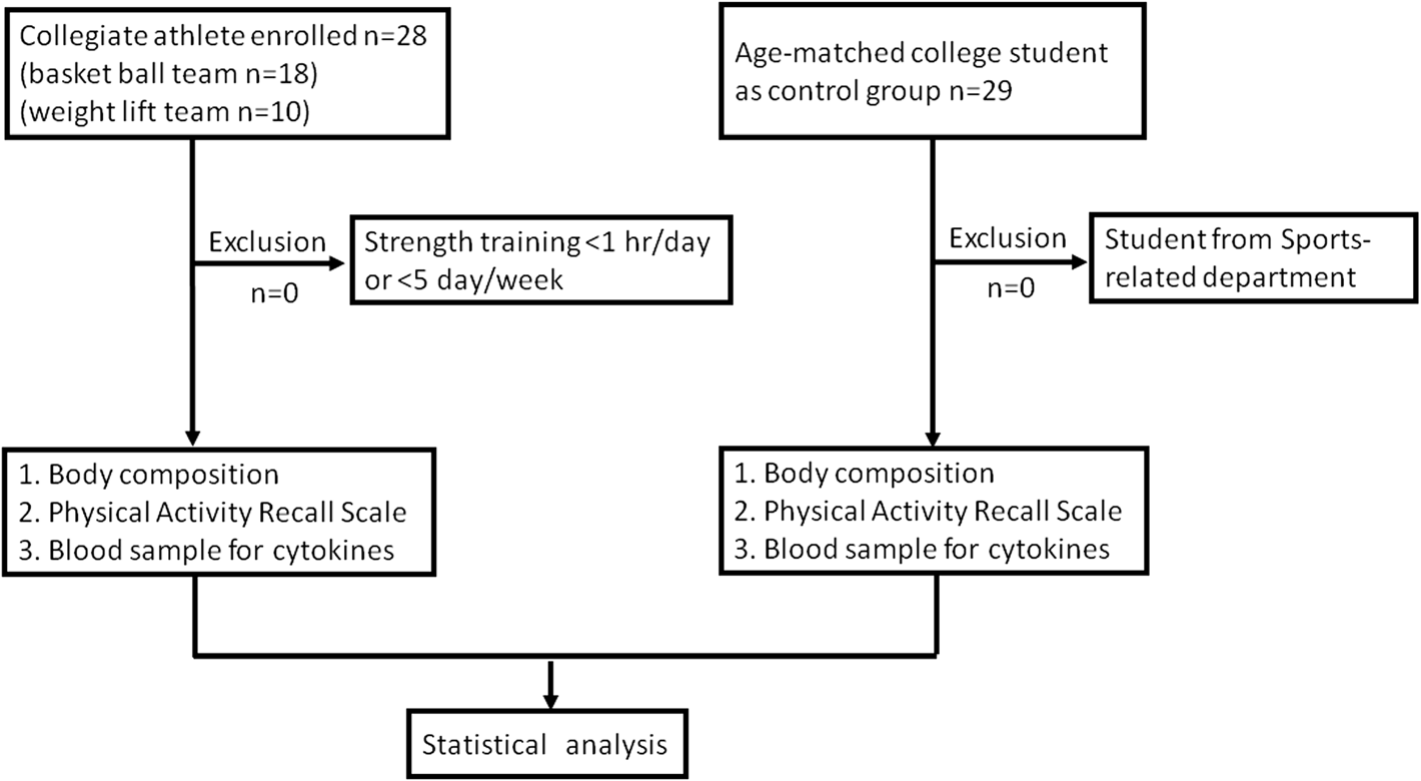
Study design

Twenty-eight male athletes from sports-related departments in the National Taiwan Sports University were recruited after obtaining their written informed consent. Their training program consisted of strength training for at least one hour per day and five times per week. Collegiate athletes were from the weight-lifting team (*n* = 10) and the basketball team (*n* = 18). Another 29 age-matched college students from non-sports-related departments were recruited as untrained controls for this study (Fig. 1). The Research Ethics Committee of National Taiwan University Hospital conforming to the Declaration of Helsinki of the World Medical Association approved the study.

Ten milliliters of whole blood sample was collected through venipuncture at antecubital vein. The serum was isolated and stored in a -20 °C refrigerator for biochemical analysis. The maximal grip strength of the dominant hand of each individual in standardized arm and hand positions was measured with an analog isometric dynamometer (Baseline® hydraulic hand dynamometer, Fabrication Enterprises Inc., Irvington, NY, USA) -the highest value of three attempts was used for analysis [16]. Height was measured to the nearest 0.1 cm and weight to the nearest 0.1 kg on an electronic scale. Waist girth was measured with a soft tape midway between the last rib and the iliac crest in a standing position. Body mass index (BMI) was calculated as weight in kg divided by the square of the height in meters.

### Body composition

The parameters of body composition, resistance, and impedance were obtained with a bio-impedance analyzer (Bio Scan 920, Maltron, UK) using the manufacturer’s suggested protocol. In brief, two injector electrodes were placed on the dorsal surface of the foot and wrist at distal metatarsals and metacarpals. Two detector electrodes were placed between the styloid process of the radius and ulna, and between the medial and lateral malleolus. During the measurement, subjects remained in a supine position with feet apart and hands at their sides. A low alternating current (500 μA, 50 kHz) was passed through the body to determine the fat-free mass, fat mass (FM), and muscle mass by the equation default in the analyzer. Percentage of body fat was calculated manually as FM/body weight × 100%. The cross-validation of this machine determining body fat was performed previously [17].

### Physical activity recall scale

The self-administered Physical Activity Recall Scale (PAR) was employed to estimate daily energy expenditure [18]. The PAR included questions about time spent sleeping and in performing moderate, hard, and very hard intensity activities each day, both weekday and weekend. The time frame measured was the 7 days previous to the interview. Total daily energy expenditure was estimated by assigning to each activity category standard values of intensity expressed as multiples of metabolic equivalents (METs). One MET was defined as the resting metabolic rate, which was approximately equal to 1 kcal/kg/h. Activities were assigned as: sleep (1.0 MET), light (1.5 MET), moderate (4.0 MET), hard (6.0 MET), and very hard (10.0 MET). By multiplying the METs and reported time spent in each activity, summing all intensities, and multiplying body weight, we could obtain a summary score for daily energy expenditure in kcal [19]. Hours spent in light activities were calculated by subtracting from 168 h the sum of the time spent in the other reported activity categories.

### Biochemistry analysis

Serum myostatin level was measured with an ELISA kit from Immunodiagnostik (Bensheim, Germany). The test employed a polyclonal antibody against a full-length myostatin peptide with a competitive immunoassay technique. The sensitivity of myostatin ELISA is 270 pg/ml, and the intra- and inter-assay variability are less than 10 and 15% [20]. Serum IGF-1 was measured with Mediagnost’s ELISA kit (Reutlingen, Germany). The sensitivity, intra- and inter-assay variabilities of the IGF-1 ELISA kit are 90 pg/ml, 6.7%, and 6.8%, respectively. The absorption of each well was read using a VersaMAX tunable microplate reader (Molecular Devices, Sunnyvale, CA, USA) at 450 nm against 620 nm as a reference.

### Statistical analysis

The test of means between the normal control and the athlete group was performed with Student’s *t* test. A Wilcoxon rank-sum test was employed to compare the means between the basketball and the weight-lifting team. Pearson’s correlation analysis was used between the serum markers and other variables. The linear regression model was used to analyze the association after adjustment between serum markers and body composition and physical activity. Then, the stepwise linear regression was used to find the determinants of myostatin and IGF-1. Statistical analysis was performed with SPSS^®^ 11.5 (SPSS Inc. Chicago, Illinois, USA) with significance levels set at 0.05.

## Results

Twenty-eight athletes and 29 age-matched normal controls were recruited. There was no difference in age between the athlete and control groups (20.5 ± 1.2 vs. 21.2 ± 1.6 years). The athlete group had greater height, weight, BMI, FM percentage, fat-free mass, muscle mass, waist girth, grip strength, and estimated daily energy expenditure. As to the biomarker level, the serum IGF-1 concentration was higher in the athlete group; however, the serum myostatin levels did not differ (Table 1). We then pooled the cohort and found the reference levels of myostatin and IGF-1 were 12.3 ± 3.6 ng/ml and 293 ± 114 ng/ml, respectively, in the young healthy males.

**Table 1:** The demographic data and baseline biomarker concentrations of college students and athletes performing strength training; data are expressed as Mean (SD)

	Control	Athlete			*P* value*
		All athletes	Basketball	Weight lifting	
N	29	28	18	10	
Age (year)	21.2 (1.6)	20.5 (1.2)	20.6 (1.3)	20.6 (1.2)	0.083
Height (cm)	174 (6)	181 (8)	185 (7)**	173 (4)	0.002
Weight (kg)	69.0 (8.9)	88.3 (15.2)	90.0 (15.4)	85.3 (15.2)	<0.001
BMI (kg/m^2^)	22.5 (2.4)	26.9 (4.1)	26.0 (3.5)	28.4 (4.8)	<0.001
Fat mass (%)	13.1 (4.4)	18.2 (9.1)	16.6 (8.1)	21.2 (10.4)	0.011
Fat-free mass (kg)	59.7 (6.1)	71.2 (7.7)	74.1 (7.2)**	65.9 (5.6)	<0.001
Muscle mass (kg)	27.8 (3.5)	34.3 (4.3)	35.7 (4.2)**	31.8 (3.4)	<0.001
Waist girth (cm)	80.2 (7.2)	89.9 (10.8)	90.0 (10.3)	89.5 (12.0)	<0.001
Grip strength (kg)	42.9 (4.4)	48.5 (6.1)	46.5 (5.6)**	52.1 (5.4)	<0.001
Myostatin (ng/mL)	12.4 (3.5)	12.1 (3.7)	12.6 (3.9)	11.2 (3.3)	0.740
IGF-1 (ng/ml)	263 (134)	324 (80)	318 (82)	334 (80)	0.043
Estimated daily energy expenditure (kcal/day)	3059 (1123)	4152 (813)	4216 (794)	4036 (877)	<0.001

*denotes *p* value of *t*-test between control and all athletes

**denotes significance of Wilcoxon rank-sums test between basketball and weight-lifting teams

Since the athlete group was composed of two sports teams, basketball and weight-lifting, we compared the difference between these two teams. The basketball team had greater height, fat-free mass and muscle mass, and less grip strength than the weight-lifting team. The two serum markers did not differ between these two sports teams, or between the control and these two teams (Table 1).

We then pooled all samples and analyzed the correlation between serum myostatin and other parameters. Myostatin correlated negatively with age and fat mass percentage. It was not correlated with muscle mass, grip strength, and daily energy expenditure. IGF-1 correlated negatively with age, and positively with height, fat-free mass, and estimated daily energy expenditure (Table 2). Myostatin was positively correlated to IGF-1, but without statistical significance. We further performed subgroup analysis to see if the correlation of serum markers and other parameters was different in the athlete group and control group, but no significant difference was found (data not shown).

**Table 2:** Correlation analysis between myostatin and IGF-1 and other parameters. The data are expressed as Pearson’s correlation coefficient (*p* value)

	Myostatin (ng/ml)	IGF-1 (ng/ml)
N	57	57
Group^a^	−0.045 (0.740)	0.269 (0.043)*
Age (year)	−0.335 (0.011)*	−0.470 (<0.001)**
Height (cm)	0.146 (0.280)	0.287 (0.031)*
Weight (kg)	−0.133 (0.324)	0.226 (0.091)
BMI (kg/m^2^)	−0.248 (0.063)	0.125 (0.354)
Fat mass (%)	−0.304 (0.022)*	0.036 (0.789)
Fat-free mass (kg)	0.040 (0.768)	0.303 (0.022)*
Muscle mass (kg)	0.024 (0.857)	0.246 (0.065)
Waist girth (cm)	−0.248 (0.063)	0.040 (0.767)
Grip strength (kg)	0.029 (0.832)	0.246 (0.065)
Estimated daily energy expenditure (kcal/day)	−0.067 (0.620)	0.380 (0.004)**
Myostatin (ng/ml)	-	0.174 (0.195)
IGF-1 (ng/ml)	0.174 (0.195)	-

*: *p* <0.05

**: *p* <0.01

^a^: Control group is 0; athlete group is 1

In order to adjust the impact of age, we employed the linear regression model to analyze the correlation after adjustment for age between biomarkers and body composition and grip strength. The level of myostatin was significantly negatively correlated with BMI, fat mass percentage, and waist girth. The IGF-1 level was correlated only with height after adjustment for age (Table 3). Based on the analysis of correlation, we then performed stepwise linear regression to find the determinants of myostatin and IGF-1. First, we took myostatin as a dependent variable, and age, BMI, fat mass percentage and waist girth as independent variables, and found that age (β = -0.897 ± 0.295, *p* = 0.004) and BMI (β = -0.268 ± 0.110, *p* = 0.018) were independently related to myostatin. Second, taking IGF-1 as a dependent variable, age, height, BMI, fat-free mass and estimated daily energy expenditure as independent variables, we found that age (β = -34.27 ± 8.89, *p* <0.001) and height (β = 360.5 ± 167.0, *p* = 0.035) were independently related to IGF-1.

**Table 3:** Regression coefficients β from multiple linear regression models for serum myostatin and IGF-1 after adjustment for age

	Myostatin (ng/ml)	IGF-1 (ng/ml)
Group^a^	−0.918 ± 0.927 (0.326)	38.3 ± 27.4 (0.169)
Height (cm)	0.051 ± 0.058 (0.377)	3.503 ± 1.673 (0.041)*
Weight (kg)	−0.042 ± 0.029 (0.149)	1.169 ± 0.869 (0.184)
BMI (kg/m^2^)	−0.269 ± 0.111 (0.018)*	1.797 ± 3.476 (0.607)
Fat mass (%)	−0.141 ± 0.058 (0.018)*	0.695 ± 1.824 (0.705)
Fat-free mass (kg)	−0.014 ± 0.052 (0.789)	2.673 ± 1.524 (0.085)
Muscle mass (kg)	−0.033 ± 0.092 (0.723)	3.502 ± 2.717 (0.203)
Waist girth (cm)	−0.085 ± 0.043 (0.050)*	0.480 ± 1.333 (0.720)
Grip strength (kg)	−0.019 ± 0.078 (0.811)	3.229 ± 2.297 (0.166)
Estimated daily energy expenditure (kcal/day)	−0.001 ± 0.000 (0.125)	0.025 ± 0.013 (0.051)
IGF-1 (ng/ml)	0.001 ± 0.005 (0.883)	-
myostatin (ng/ml)	-	0.600 ± 4.072 (0.883)

Data are expressed as β ± standard error (*p* value)

*: *p* <0.05

^a^: Control group is 0; athlete group is 1

## Discussion

In this study, we established the reference value of myostatin and IGF-1 and found their relationships with body composition from a cross-sectional cohort. We demonstrated that the reference value of the myostatin serum concentration in healthy young males was 12.3 ± 3.6 ng/ml, which was correlated negatively with age. After adjustment for age, the reference value was negatively correlated with BMI, fat mass percentage, and waist girth. The reference value for IGF-1 was 293 ± 114 ng/ml, which was correlated with age negatively and height positively.

Because myostatin is thought to be a myokine—a muscle secreted cytokine [12], its serum levels have been reported in clinical conditions related to muscle growth rather than in the healthy condition. However, it is difficult to compare the serum myostatin level with previous studies, since the reported levels varied greatly due to the use of different ELISA kits. Using the same ELISA kit as in our study, Ju and Chen revealed that the serum myostatin level was higher in patients with chronic obstructive pulmonary disease (COPD) than in the healthy control (11.85 ± 4.01 vs. 7.46 ± 2.21 ng/ml) [21]. The myostatin level of COPD patients was correlated with BMI negatively and with the serum tumor necrosis factor α level positively. Using the same ELISA kit, Zamora et al. found the mean myostatin serum concentration was 12.3 ng/ml in chronic heart failure patients aged 72.3 years on average [22]. No correlation was found between serum myostatin and parameters of disease severity and prognosis. Wintgens et al. demonstrated that the myostatin level was elevated in decompensated congestive heart failure patients compared with the normal control (49 vs. 32 ng/ml). The level decreased to the normal level after recompensating therapy [20]. Lakshman et al. measured serum myostatin level with a monoclonal antibody-based sandwich ELISA. They reported the myostatin level to be 8.0 ± 2.3 ng/ml in healthy young men with an average age of 26.5 years [23]. The level in older men with age of 66.4 ± 4.7 years dropped significantly to 7.0 ± 2.5 ng/ml. In summary, the serum concentration of myostatin seems to range from 7 to 32 ng/ml in the general population.

Myostatin affects the growth of both muscle and lipid. Our study, recruiting healthy young male subjects, found myostatin was negatively related to BMI, fat mass percentage, and waist girth after adjustment for age. Feldman et al. reported that myostatin transgenic mice had immature adipogenesis resulting in lower body weight, serum triglycerides (TG), and fasting plasma glucose [24]. Two other studies also concluded that administration of recombinant myostatin protein to 3 T3-L1 pre-adipocytes or human mesenchymal stem cells blocked their adipogenesis [25]. In a human study, a 6-month lifestyle intervention reduced the body weight of obese children aged between 6 – 16 years. At the same time, serum myostatin elevation and a decrease in fat mass and BMI were also observed [26]. Furthermore, in a genetic study recruiting 500 non-diabetic Asian Indians in north India, subjects with myostatin polymorphism A55T were reported to have a higher body fat percentage, fat mass, and truncal adiposity [27]. We have observed that a lower serum myostatin concentration is related to metabolic syndrome, central obesity, low high-density lipoprotein cholesterol, and high TG among 246 diabetic patients after adjusting age and gender [28]. Thus, when we determine the normal value of serum myostatin, it is prudent to take fat-related parameters into consideration.

Myostatin exerts its main effect through autocrine/paracrine mode, and excess myostatin peptide will spill-over into the circulation. Like the other members of the TGF-β superfamily, myostatin is regulated by complex interactions with many proteins in extracellular matrix [29]. Therefore, intracellular mRNA and secretion in isolated muscle tissues may not faithfully reflect its circulating levels. Matsakas et al reported that rat myostatin mRNA expression reduced after swimming training for 4 weeks, not after acute bout of exercise; however, no myostatin protein change was detected [30]. *Myostatin is also expressed in the cardiac muscle, and this may contribute to the serum myostatin level* [31]. Myostatin mRNA expression is increased after infarction and exercise in the cardiac muscle, but remain unchanged in the canine model of dilated cardiomyopathy and chronic heart failure [22, 32]. Myostatin may be a mediator of cardiac development; however, the exact mechanism still need to be elucidated [33].

We previously proposed an “accelerator-brake model” to illustrate the role of myostatin in regulating muscle growth [12]. Myostatin served as a negative feedback molecule during the process of muscular growth or regeneration and limited the skeletal muscle mass [10]. In this model, myostatin is activated only when muscle is growing or regenerating. Our athletes performing strength training had significantly greater muscle mass, BMI, grip strength, and daily energy expenditure than their untrained counterparts. However, the serum myostatin level of the two groups was not significantly different. The main problem might be the timing of blood sampling after exercise training. In another study, the acute response to a single bout of isometric exercise in rats was a rapid increase in myostatin mRNA levels 30 min to 6 h post-exercise, and a return to baseline levels 24 h post-exercise [34]. The athlete’s body composition is at a steady state, so there is no difference in myostatin concentration. Future longitudinal study to observe the dynamic change of muscle mass after strength training may prove the role of myostatin in decelerating muscle growth.

Age is a determining factor of serum myostatin and IGF-1. Lakshman et al reported lower myostatin level in the elderly population aged 66.4 ± 4.7 years [23], which could be explained by “accelerator-brake model”. The negative relation between IGF-1 and age was also well established [35]. Since different age groups have different muscle mass, it is desirable to establish an age-specific norm for the level of these biomarkers. However, the age range of our subjects is too narrow to establish such a norm, so we tried to show their relationship with linear regression model.

IGF-1, a downstream molecule of growth hormone, is a serum protein with insulin-like metabolic activities and growth controlling ability. IGF-1 expression activates muscular satellite cells and counteracts muscle function decline in the mouse model of muscular dystrophy [14]. GH or IGF-1 has been taken by athletes for its anabolic effect to enhance sports performance [36]. Since the concentration of GH is highly variable due to its pulsatile secretion pattern, IGF-1 is suggested as a good indirect surrogate for the GH/IGF-1 axis in the GH-2004 Project. An elevated serum IGF-1 level indicated sustained activation of the GH-IGF-1 axis. The reported normal value of IGF-1 is 297 ± 10 ng/ml, and is related to gender, age, nutrition status, and GH administration [35]. In our study, the serum IGF-1 level was 293 ± 114 ng/ml, which was comparable to previous published reports. Future evaluations of serum IGF-1 levels in other sports are crucial for application in anti-doping.

There are some limitations in this study. First, the sample size was small, so the statistical power was limited. Besides, measurement errors in self-reported physical activity levels might interfere with regression analysis. Although PAR is accurate in estimating energy expenditure compared with other physical activity questionnaires [37], subjectively perceived exercise intensity may vary between individuals. Furthermore, the MET value assigned to each intensity level of physical activity may differ in respondents having different BMI [38]. In addition, since the reference value is only for male athlete in certain sports, further validation in other sports is needed for actual implementation. Finally, because no commercial kit was available, we cannot measure the concentration of the pro-peptide, which inactivates myostatin by binding on it [4].

## Conclusions

In conclusion, the reference values of serum myostatin and IGF-1 levels were 12.3 ± 3.6 ng/ml and 293 ± 114 ng/ml, respectively, in the young healthy males. The serum myostatin level was negatively related to body fat percentage; and serum IGF-1 was positively related to height after adjustment for age. It is necessary to detect serum myostatin together with other biomarkers, such as IGF-1, follistatin, and propeptide. Measurement of myostatin alone would not be sufficient. The reference values can provide a basis for future study, especially in the field of doping.
